# Augmenting Traditional Support Groups for Adolescents With Type 1 Diabetes Using Instagram: Mixed Methods Feasibility Study

**DOI:** 10.2196/21405

**Published:** 2021-10-21

**Authors:** Faisal S Malik, Cara Lind, Sarah Duncan, Connor Mitrovich, Michael Pascual, Joyce P Yi-Frazier

**Affiliations:** 1 Department of Pediatrics University of Washington Seattle, WA United States; 2 Seattle Children's Research Institute Seattle, WA United States

**Keywords:** diabetes mellitus, type 1, self-help groups, social media, adolescent

## Abstract

**Background:**

In-person support groups have been shown to benefit adolescents with type 1 diabetes (T1D) by helping to decrease perceived diabetes burden and improving knowledge related to chronic disease management. However, barriers exist to participation in traditional support groups, including the timing and location of meetings and resources needed to attend. Adolescents are increasingly utilizing online support groups, which may provide solutions to some of the challenges faced when implementing in-person support groups.

**Objective:**

The purpose of this study was to assess the feasibility and acceptability of a hybrid support group model where traditional in-person support groups were augmented with Instagram participation between monthly support group sessions for adolescents with T1D.

**Methods:**

Participants (13-18 years old with T1D for ≥6 months) were asked to post photos each week for 3 months based on predetermined topics related to diabetes management. At the end of each month, participants attended an in-person support group to discuss their photos using the Photovoice method. Feasibility was assessed through enrollment and retention, number of Instagram posts, poststudy questionnaire, and a template analysis of the focus groups.

**Results:**

Of 24 eligible participants, 16 (67%) enrolled in the study, with 3 dropping out prior to support group participation. The number of photos posted over 3 months ranged from 14 to 41. Among the 11 participants who completed a follow-up questionnaire, the majority of participants (6/11, 55%) reported that they very much enjoyed participating in the hybrid support group, and more than three-quarters (9/11, 82%) of participants reported that they “related to the photos posted.” Over half of participants (8/11, 73%) reported “learning something new from the photos posted,” which arose from sharing knowledge and experiences related to navigating the common challenges of diabetes management. Additionally, the use of Instagram posts helped facilitate peer discussions during the in-person support groups.

**Conclusions:**

The novel combination of using Instagram to augment traditional in-person support groups was feasible and acceptable to adolescents with T1D. The overall satisfaction with the hybrid support group model, combined with the observed engagement with peers between support group sessions over social media, suggests that a hybrid support group model may have the potential to provide more pronounced benefits to adolescents than in-person meetings alone. Future research should investigate the use of social media as part of the support group model and examine the potential improvement of self-esteem, benefit-finding, and social support using validated tools in adolescents with diabetes.

## Introduction

Adolescents with type 1 diabetes (T1D) struggle with difficult and complex treatment plans to maintain adequate glycemic control. Heightened concerns about social context and peers, the premature shift in responsibility for management from parent to adolescent, fatigue from daily diabetes care (ie, diabetes burnout), and incomplete knowledge and understanding of treatment regimens and future health risks have been cited as barriers to diabetes care in this age group [1,2]. As a result, only a small proportion of adolescents with T1D meet targets for glycemic control [3], placing them at higher risk for long-term microvascular and macrovascular complications [4,5].

Although pubertal factors may partly contribute to poor glycemic control in adolescence, psychosocial factors consistently demonstrate meaningful associations with glycemic outcomes [6,7]. Youth with diabetes have a greater incidence of depression and psychological distress compared to their healthy peers [8]. Depression and distress have been associated with worse glycemic control, more complications, higher health care costs, and increased frequency of adverse events [9]. The high rates of distress and depression coupled with poor outcomes in adolescents with T1D highlight the need for age-specific preventative interventions.

Previous studies have shown that adolescents with T1D who participate in support groups have significantly less perceived diabetes burden and more knowledge of the disease [10-12]. Moreover, support groups that provide adolescents with T1D with coping skills training and peer support lead to improved adjustment and metabolic control [13,14]. However, there are often difficulties implementing and maintaining these types of traditional support groups due to location, possible interference with school demands, and resources needed to attend [15,16].

Today, it is common for youth to look to online communities and social media for support and guidance [17,18]. Indeed, online support groups provide an opportunity to overcome some of the limitations related to traditional in-person support groups, particularly related to resources and engagement issues [19,20]. Therefore, this study aimed to explore the feasibility and acceptability of augmenting traditional in-person support groups with Instagram support between monthly support group sessions for adolescents with T1D.

## Methods

### Participants

Eligible participants were 13 to 18 years old with a diagnosis of T1D for at least 6 months from existing clinic patients at Seattle Children’s Hospital (SCH). Participants were required to be English-speaking and have personal access to Instagram, a popular social networking photo application, through their smartphone. At the time of the study, Instagram was available on Android (version 2.2 or above) or Apple iOS devices (version 4.2.1 or above) with a camera attached (iPhone 3GS or above; iPod Touch with an internet connection, 3rd and 4th generations). We excluded any adolescents with a major psychological or psychiatric disorder based on social work notes from the previous year.

The protocol was approved by the Seattle Children’s Research Institute Institutional Review Board, and voluntary informed written assent and consent were obtained from each participant and their caregiver (for those <18 years old). A total of 16 adolescents agreed to participate in the study.

### Study Protocol

At the first visit, after informed assent and consent were obtained, the participant was given basic training on using and sharing content on Instagram. This training included instructions and tips on downloading the application onto the participant’s phone, adjusting privacy settings on Instagram, sharing photos, interacting with others through the app, and allowing the study team to follow their account. Real names were not required to be used for privacy purposes. Current Instagram users were given the option of using their account or setting up a new one for the study, with privacy considerations discussed in each case. Participants were encouraged to “follow and friend” their cohort members, but this was not required. The study staff had their own Instagram account, and the participants were required to allow this account to “follow” the user.

Participants were placed into one of two cohorts. Placement into each cohort was determined solely by time of consent: the first half enrollees were assigned to the first cohort, and the second half were assigned to the second cohort. The intervention consisted of two main components. The first was sharing photos with their cohort on Instagram either through their general Instagram feed or through “direct messaging.” All participants were provided the list of the other cohort members’ Instagram names during the first week. Topics of photos were suggested each week to aid participation (Textbox 1). These topics were generated from previous work in this area [21]. When posting photos, participants were encouraged to caption their photo and use the study-specific hashtag to ease the identification of photos related to this project.

**Suggested Instagram photo feed topics.**

**Month #1**

- Week 1: What I eat
- Week 2: How I stay active
- Week 3: What I find funny
- Week 4: Free choice

**Month #2**

- Week 1: Struggles and challenges
- Week 2: What makes me happy
- Week 3: How I cope
- Week 4: The truth about diabetes

**Month #3**

- Week 1 How others view my diabetes
- Week 2: What I can teach others
- Week 3: What I have learned
- Week 4: Free choice

A monthly support group meeting was scheduled at the end of each month.

The second component of the intervention was participation in an in-person support group at the end of each month. Support groups were conducted using the Photovoice method, a community-based participatory research method in which participants take photos about a community issue and then discuss the photos in a group setting where they can reflect on these experiences and issues and feel empowered to make changes [22,23]. The Photovoice method has been shown to improve meaning-making, life satisfaction, and empowerment in many diverse populations and has also been shown to provide psychosocial support for various adolescent communities [24-26].

Support groups were held in approximately 1-hour sessions at SCH by cohort during an evening or weekend every 4th week of the intervention. In preparation for the discussion, study staff printed photos posted that month. A study staff member trained in facilitating support groups used the SHOWeD approach to guide the group sessions. The SHOWeD method focuses on five questions: (a) what do you see here, (b) what is happening here, (c) how does this relate to our lives, (d) why does this issue exist, and (e) what can we do about it [22]? Discussion tactics included pile sorting, categorization of topics, and other techniques garnered from traditional Photovoice methodology [27]. Feedback about comfort and satisfaction with the intervention was also discussed. Support groups were recorded and transcribed for the template analysis. At the end of the study, participants were asked to complete a follow-up questionnaire that assessed the acceptability of the intervention. Participants were given a US $20 gift card for each group attended and a US $10 gift card if all follow-up surveys were completed.

### Feasibility Measures

#### Acceptability

Acceptability was assessed through enrollment and retention rates and survey questions evaluating the acceptability of the intervention. These questions included overall satisfaction of the intervention, how interesting the photos were that were shared with listed options (“very interesting,” “interesting,” “mildly interesting,” and “not at all interesting”), whether they could relate to the photos they saw, whether they learned anything from the photos shared, and their comfort with posting and privacy issues. In addition, participants were given room to give comments and suggestions and to expand on their responses.

#### Implementation

Implementation was evaluated by examining support group attendance and the actual use of Instagram during the intervention, including the number of posts and likes by study participants. In addition, a template analysis of the support groups was carried out [28]. For the template analysis, support groups were digitally recorded, transcribed verbatim, and reviewed by investigators to ensure data integrity. A hierarchically organized codebook was developed a priori based on the feasibility domains of acceptability and implementation. Support group transcripts were coded by 2 team members using the final version of the codebook and Excel (2019; Microsoft Corporation). Coders were blind to each other’s coding, and all differences were resolved by discussion with a third team member until 100% agreement was reached. During synthesis, coded excerpts were summarized into theme domains related to feasibility with associated quotes. In addition, Instagram posts related to themes were identified.

## Results

### Acceptability

Adolescents in this study provided insights into the potential acceptability for the hybrid support group model. Participants also expressed to what extent the intervention was suitable and satisfying to recipients.

#### Rates of Enrollment and Retention

We approached 24 English-speaking, 13 to 18-year-old patients with T1D for at least 6 months to participate in this study. Of the 24, 16 (67%) enrolled (Table 1). Reasons shared for not enrolling included no personal access to Instagram (4/24, 17%), no interest (2/24, 8%), and being too busy (2/24, 8%). All 16 enrolled participants reported using Instagram prior to enrollment, and they were divided into two equal cohorts (8 participants each), of whom 2 (13%) elected to create new Instagram accounts for study participation. Of the 16 who enrolled, 3 (19%) participants from the second cohort dropped out after enrollment but prior to participation. Among the remaining participants, 85% (11/13) that engaged in at least one support group completed the acceptability questionnaire at the end of study participation.

**Table 1:** Demographic and clinical characteristics for enrolled adolescent participants (n=16).

Characteristics		Value
Age (years), mean (SD)		15.3 (2.3)
**Gender, n (%)**		
	Female	9 (56.25)
	Male	5 (31.25)
	Prefer not to answer	2 (12.50)
**Race/ethnicity, n (%)**		
	Non-Hispanic White	10 (62.50)
	Other	4 (25.00)
	Prefer not to answer	2 (12.50)
**Health insurance, n (%)**		
	Private	9 (56.25)
	Public	3 (18.75)
	Not sure or prefer not to answer	4 (25.00)
Baseline HbA_1c_ (%), mean (SD)		9.2 (2.3)
Diabetes duration (years), mean (SD)		5.3 (4.4)

#### Satisfaction

Of participants that completed the acceptability questionnaire, 55% (6/11) reported that they “very much enjoyed” participating, 45% (5/11) reported that “it was OK,” and none stated that they “thought they would enjoy it more” or “did not like it at all.” When asked how they would rate the suggested topics, one participant rated the topics “very interesting,” while all others rated them “interesting.” No one endorsed “mildly” or “not at all” interesting.

For the majority of participants, the ability to reflect and relate to photos from other adolescents with T1D was a major draw to engage in the in-person support groups. Most participants reported that the ability to learn from others and not feel alone in navigating the daily diabetes self-care challenges was a key reason they enjoyed posting and participating in the support groups.

Just feeling like you’re not the only one going through it…and that you can still lead like a normal life and still have diabetes. Plus, [seeing] other people also going through the struggles so you aren’t there alone.

Some also touched on how they found the hybrid support group model more empowering than other group settings they had participated in, such as diabetes camps.

I’ve only had [T1D] for 2 years, so I feel like it was more helpful for me. Some of the posts and the pictures were [about] things that I didn’t really know because I don’t really usually talk to other people about [my] diabetes. Because I think going to [diabetes] camps, like they say they’re supposed to make you feel better but whenever I try to talk, it just makes me feel worse.

### Implementation

Participants also shared their views on the successful execution of using Instagram as a modified application of Photovoice and support group participation. Specifically, adolescents offered insights into the extent, likelihood, and manner in which the hybrid support group model can be fully implemented as planned and proposed.

#### Support Group Participation

Attendance rate of support groups varied over 3 months. In the first cohort (8 participants), 75% (6/8) participated in the first support group, 63% (5/8) in the second, and 38% (3/8) in the final support group. In the second cohort (5 participants), 60% (3/5) participated in the first support group, 100% (5/5) in the second, and 80% (4/5) in the final support group. Many participants shared how they felt the support groups were really beneficial in augmenting the self-reflection and discussion of photos on Instagram.

[The support groups] were really beneficial to, like, see other people’s views and stuff about it. For me, I didn’t really look at other people’s pictures when they were posting, I just thought about mine and then moved on because I had other things to do.

Participants also shared that the in-person support group helped them connect more effectively with group members online, as they knew who they were interacting with on Instagram.

I think that [meeting participants in the support groups] pushed me to post more…I know like the people and their faces so I’ll be, ‘Oh, I know that person kind of, like I’ve seen her.’

I liked being able to put the posts with the people. Because like seeing your posts I didn’t get a ton out of your personalities but then coming here I was able to meet people.

### Using Instagram as a Modified Application of Photovoice

**Figure 1:**
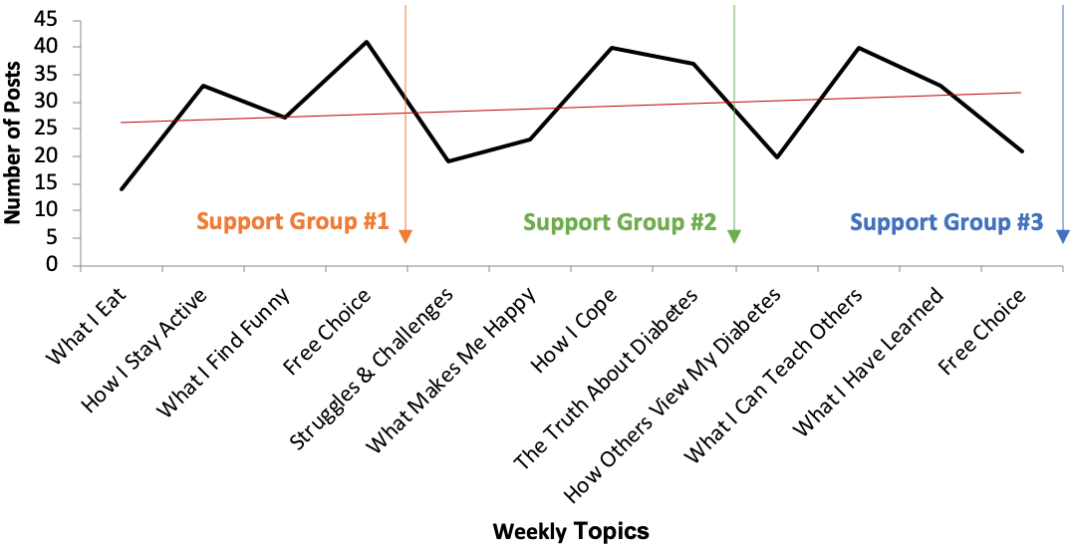
Number of Instagram posts between support groups.

Participation by week is represented in Figure 1. The linear trend line shows an average upward trend in the number of posts throughout the study. Between both cohorts, the number of photos posted ranged from 14 to 41, with the highest participation charted for the first “free choice” (no suggested theme), “how I cope,” and “what I can teach others” topics. The lowest number of posts were seen with the suggested topics of “what I eat,” “struggles and challenges,” and the “free choice” topic suggested the second time.

**Figure 2:**
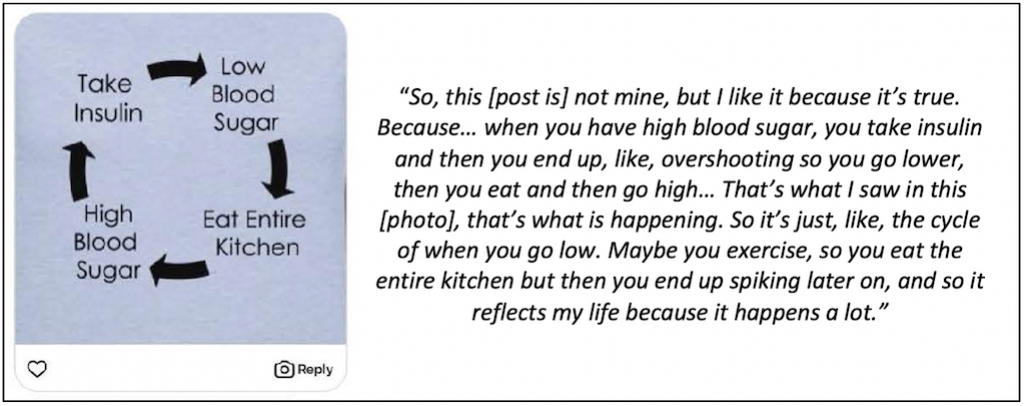
Example post of a participant “relating to the Instagram post” at a support group.

The majority of respondents (7/11, 64%) reported their cohort members’ photos as “very interesting.” The remaining participants described their cohort members’ photos as either “interesting” or “mildly interesting.” No participants marked “not interesting.” In addition, 82% (9/11) of participants reported “relating to the Instagram posts” (Figure 2).

**Figure 3:**
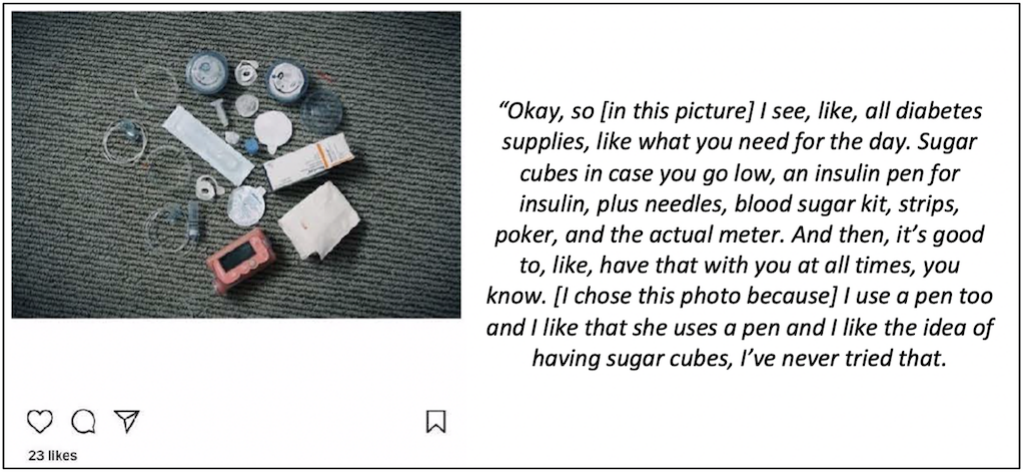
Example post of a participant “learning something new from the photos posted” at a support group.

Moreover, 73% (8/11) reported “learning something new from the photos posted” (Figure 3). When asked to expand on what they learned, participants indicated posts that included suggestions on how to make healthy snacks, how to think about the impact of different types of food on glycemic control, and how other youths with T1D experience similar struggles with diabetes management were particularly helpful.

**Figure 4:**
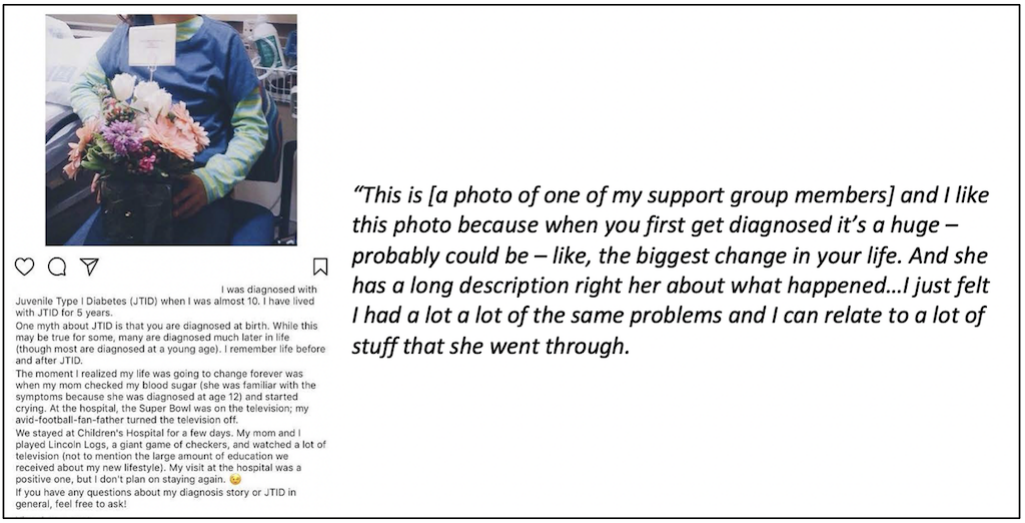
Example post with text caption facilitating peer discussions at a support group.

In addition to posting photos, most Instagram posts by participants included text captions, which routinely provided context for the photos posted. Captions accompanying the posts provided the participants with the ability to share personal stories and experiences about particular issues. Posts with captions facilitated peer discussions during the in-person support groups (Figure 4).

None of the participants who completed a follow-up survey reported any privacy concerns before the study, during participation, or after the study.

## Discussion

### Principal Results

We found that the novel combination of using Instagram to augment traditional in-person support groups was feasible and acceptable to adolescents with T1D. Participants posted Instagram photos consistently between support group visits and found other members’ photos interesting and relatable. Although in-person support group attendance varied over 3 months and between cohorts, no participants reported dissatisfaction with the hybrid support group intervention. Given the need for increased support for adolescents with T1D and the overall feasibility and acceptability of this intervention, combining traditional in-person support groups with online support group options through social media use could be a means to increase engagement in psychosocial support outside of the clinic setting.

Our study enrollment rate for this feasibility study was comparable to traditional in-person support groups for adolescents [29,30]. Additionally, participants’ attendance and satisfaction were similar to our previous study in which we assessed the feasibility and acceptability of using Instagram to implement the Photovoice method to share diabetes-related information [21]. By offering the social media component between support groups, participants were given increased opportunities to engage in photo-sharing and discussions of weekly topics than what is offered in traditional Photovoice projects.

The overall satisfaction with the hybrid support group model also highlights the potential for more pronounced benefits using the Photovoice method than what is currently seen from in-person support groups alone. Given that a higher level of emotional support from peers is predictive of less diabetes-related distress [12], strategies to promote positive peer interactions are needed. Photovoice, rooted in core community-based participatory research principles, stresses empowerment and emphasizes individual and community strengths, colearning, and community capacity building [31]. Our findings demonstrate that adolescents benefited from self-reflection and discussion of photos in the support group sessions using the SHOWeD questioning technique. In addition, the finding that a majority of participants reported learning new approaches to support diabetes self-care and management reinforces the value of support groups in promoting peer education.

In our study, participants remained engaged in the support group topics even outside of in-person sessions. Support groups have been shown to increase self-care behaviors and decrease the perception of diabetes-related burden in young adults with T1D [11]. However, these benefits are difficult to sustain if the educational and psychosocial elements provided from involvement with support groups are not continuously reinforced [32]. The addition of social media with in-person support groups, as shown in our study, could be a means to facilitate reinforcement between support group sessions. While in-person support group sessions are held at set times, which members may or may not be able to attend depending on their schedule, social media can be accessed at any time of the day and for any number of times depending on the user’s needs.

Of additional interest, some participants shared that connecting with Instagram community members in person motivated them to engage more online after support group participation. Patients with diabetes are increasingly looking to online communities on the internet for clinical information and to provide and receive support [33]. While utilized by many, the helpfulness of online support groups varies depending on the media platform, intervention style, and target population, and its effectiveness has yet to be accurately identified [34,35]. Furthermore, the authenticity of online group members and the information that they share may not always be trustworthy [36]. The hybrid support group model used in this study could address some of these concerns by allowing participants the opportunity to verify that the Instagram profiles are for the same individuals they met in their support group sessions.

### Limitations

While this study provides insight into the acceptability and feasibility of a hybrid support group model, it has limitations. The first is the small sample size due to the pilot nature of the study, which limits generalizability. The second limitation is that we may not have fully replicated a typical Instagram social media environment for the 2 participants that elected to create a separate Instagram account for the study. Those who created a separate account might not have had as much engagement in their study-specific account, likely due to the inconvenience of switching between their personal account, where perhaps most of their consistent social media engagement is, and their new study account to meet the weekly posting suggestions. Third, we were unable to capture participant interaction that could have been taking place via direct messaging or other social media platforms that may have provided additional insight into participant engagement between monthly support groups. Finally, given that the majority of our participants used their primary Instagram account for study participation, it is possible that some participants self-censored the content of their diabetes posts since it was viewable by their other nonstudy Instagram followers. However, the potential utility of the hybrid support group model would remain, given that the majority of participants posted reliably and engaged with support group peers despite having nonstudy participants also view their social media content.

### Conclusions

Adolescents with T1D often struggle with managing their disease, and as a result, they may look online or offline for the psychosocial support they need. Traditional in-person support groups provide many benefits, but some barriers prevent many youths from engaging. Online support groups are easily accessible for many adolescents and offer them the ability to connect with and learn from others that they may not have otherwise been able to; however, the authenticity of their online peers may not always be trustworthy. Our study demonstrated that a hybrid combination of in-person and online support groups is feasible and acceptable, offering the potential for increasing social support and optimizing diabetes outcomes in young adults with T1D. Future studies should focus on examining the amount of in-person versus online support required to support adolescents with T1D, along with the efficacy of this hybrid support group model, including its impact on diabetes outcomes and self-esteem, benefit-finding, and social support using validated tools in adolescents with diabetes.

## Abbreviations

T1D: type 1 diabetes
SCH: Seattle Children’s Hospital
NIH: National Institutes of Health
